# Necrosis

**Published:** 1880-06

**Authors:** H. H. Townsend

**Affiliations:** Pontiac, Ill.


### ARTICLE VI.

### *Necrosis.*

BY H. H. TOWNSEND, PONTIAC, ILL.

On Saturday, September 7th, 1878, Mrs. R., about forty years of age, acting as a commercial traveler, came to consult me in regard to what she called an "ulcerated tooth." I was at once impressed with her feeble and emaciated look, which very much resembled a patient well advanced in consumption. The red blood corpuscles seemed sadly deficient. Pulse feeble and very frequent, something over 100. The history of the case, as nearly as I could get it, was, that some two months previous, she had had some teeth filled with Amalgam, and shortly afterwards began to notice a coppery taste in her mouth, smarting of the throat and fauces, soreness of all the teeth, and increased flow of saliva—the symptoms increasing in severity until profuse salivation ensued. This lasted some days, and after it subsided an abscess broke over the labial surface of the right superior cuspid root. Her dentist called it a case of "ulcerated tooth," and singularly enough, treated it through the fistula some six or seven weeks without removing the filling to ascertain the condition of the pulp. When I saw the case there were several fistulous openings discharging thick yellow pus. On examination with a probe I found the outer alveolar plate necrosed from near the first bicuspid nearly to the lateral incisor, and extending upward about one-fourth inch beyond the apex of the cuspid root.

Exfoliation had already taken place, and a few incisions through the gum with a lance, enabled me to remove the sequestrum with very little difficulty. The hemorrhage was slight, requiring no treatment. The wound was syringed with tepid water containing about four drops of creasote and one grain of morphine to the ounce. As the inflammation had extended laterally to the first molar on the right side and to the first bicuspid on the left, and the patient being in such an anæmic condition, I was apprehensive that the necrosis would also extend and result in the loss of, perhaps, the entire jaw. I therefore painted the gums as far as inflamed, with tr. aconite root and tr. iodine, equal parts, with a view of allaying the inflammation and if possible preventing any further spread of the disease.

Prescribed a tonic of quinine and iron, as follows: Tr. muriate of iron, 1 oz.; sulphate of quinine, 1 dram; water to make 4 oz.; teaspoonful in water three times a day. Also wine and egg before breakfast; rare beef-steak, graham, and fruit; recommended as a diet also milk and egg. I removed the filling from the cuspid and found that the pulp had never been exposed, and was alive and probably in a healthy condition. I determined if possible to save this tooth, notwithstanding it had no bony covering on its labial surface. The cavity was filled temporarily with gutta-percha, and the patient dismissed at 12 M., with instructions to call again at 6 P. M. Came promptly on time. Syringed as before, and repeated the application of aconite and iodine. Saw the patient again Sunday morning. Had slept well and was much refreshed. Color improving. Pulse 80 and stronger. Inflammation of the gums subsiding. Discharge of pus considerably diminished. Treated as before, and repeated it again Sunday evening.

Monday morning found considerable improvement in every respect. Syringed with an alcoholic solution of salicylic acid in water. Applied the aconite and iodine as before, and repeated the treatment again at 4 P. M., as she was on her way to the train to leave town. The improvement was all that could possibly be expected in so short a time. Pulse strong and natural. Very little discharge of pus, and the inflammation of the gums very much reduced. As her business took her to Peoria, Ill., I sent her to my friend, Dr. J. M. Hunt, of that city, giving her a letter of introduction, containing a brief history of the case and my treatment. Some two weeks later I received a letter from

Dr. Hunt, stating that he had continued the treatment except the application of the aconite and iodine, which he had discontinued a few days previous, as it was no longer needed. Reported the case doing finely. The deposit of new bone being quite perceptible. How long she remained there I do not now remember, but after leaving Peoria she syringed it herself, using the salicylic acid water for the purpose. Saw the patient again the next April, and found the reproduction of bone had been complete, except at a point just above the termination of the enamel on the labial surface of the cuspid, where the gum had receded, leaving a little notch in the gum. In December, 1879, I removed the gutta-percha filling from the cuspid and re-filled with gold. The pulp was alive and healthy, and the tooth as firm in its socket and as useful as any of its neighbors.

Now, as to the cause of the necrosis. My own opinion is, that the periostitis accompanying the salivation was the exciting cause, the debilitated condition of the patient tending to an unfavorable termination. What produced the salivation is not so clear. As it occured soon after the introduction of some amalgam fillings, without the use of the rubber dam, and as the patient declared that she had taken no medicine whatever, I thought perhaps some particles of the filling had been swallowed, containing sufficient mercury to salivate. My theory of this however was somewhat shaken by learning of a case of salivation following the insertion of an amalgam filling in my own practice. On the 24th of August, 1878, I filled a mesial cavity in a right superior second molar, for a farmer in good health. The cavity was a simple one, not extending to the grinding surface. The first molar was missing, affording easy access to the cavity, which was prepared, filled and finished with the rubber dam on the tooth. The amalgam used was one of the gold and platinum alloys in the market, and was used as dry as it could be worked, and tin foil pressed upon the surface of the plug, to absorb any surplus mercury that might remain. The filling was burnished before the dam was removed, and I *know positively* that not one particle of the filling was swallowed. Yet, in a few weeks, the patient returned, complaining that he had been badly salivated soon after his tooth was filled. I examined the filling carefully, and found it in perfect condition. Also questioned the patient closely, and he insisted that he had taken no medicine of any kind. I have no theories to offer. Since writing the above, I have received a statement from Dr. Hunt of his treatment of the case of necrosis, its condition, progress, &c., which I append, as follows:

Mrs. M. H. R. came to my office in September, 1878, upon recommendation of Dr. H. H. Townsend, of Pontiac. Upon examination of the right superior lateral incisor, cuspid, and first bicuspid, I found the incisor and bicuspid slightly loose, but the cuspid was in a much worse condition, being affected by a lesion of the gum tissue on the mesial side, presenting an opening about half an inch deep, by about an eighth of an inch in diameter at the orifice, extending alongside the root toward the apex. The mucous membrane over the adjacent teeth presented a healthy hue, but over the cuspid, an appearance slightly purple, as though nature was timid in her expression of an effort she was being urged to make in a recovery of the gum tissue to a physiological condition. There was an appearance of partial recovery from necrosis, leaving a deep, well-defined sinus, with a constant, slightly appreciable deposit of granulations at its apex. The objects had been to prevent a union of the lips of the orifice, next to invite continued granulations of the deepest portion of the sinus, and lastly, to bring about a healthy color of the gum tissues. To this end I continued the solution of salicylic acid, as recommended by Dr. Townsend, as being the best remedy, in my opinion, to invite granulations from the farthest point of the sinus, and preventing a closure of the orifice, and made a topical application with aconite and iodine; directing the lady to continue Dr. T.'s constitutional treatment.

When the lady left me in six or eight weeks, the incisor and bicuspid were as firm as the other teeth, and the cuspid was much firmer than when I first saw her. I had the pleasure of seeing the case some months subsequently, and the granulations were making rapid progress toward a completion of the ultimate desire. The cuspid was firm, but not as firm as the other teeth, still very encouragingly so, much to my gratification, and thanks to Dr. T., and Mrs. R., who so kindly seconded my best feeble efforts towards the conclusion which I am now glad has been reached.—*Dental Advertiser.*

